# Natural Killer Cells as Key Mediators in Type I Diabetes Immunopathology

**DOI:** 10.3389/fimmu.2021.722979

**Published:** 2021-08-20

**Authors:** Graeme Gardner, Christopher A. Fraker

**Affiliations:** Tissue and Biomedical Engineering Laboratory, Leonard M. Miller School of Medicine, Diabetes Research Institute, University of Miami, Miami, FL, United States

**Keywords:** natural killer cells, type 1 diabetes, beta cell, immunopathology, autoimmune

## Abstract

The immunopathology of type I diabetes (T1D) presents a complicated case in part because of the multifactorial origin of this disease. Typically, T1D is thought to occur as a result of autoimmunity toward islets of Langerhans, resulting in the destruction of insulin-producing cells (β cells) and thus lifelong reliance on exogenous insulin. However, that explanation obscures much of the underlying mechanism, and the actual precipitating events along with the associated actors (latent viral infection, diverse immune cell types and their roles) are not completely understood. Notably, there is a malfunctioning in the regulation of cytotoxic CD8+ T cells that target endocrine cells through antigen-mediated attack. Further examination has revealed the likelihood of an imbalance in distinct subpopulations of tolerogenic and cytotoxic natural killer (NK) cells that may be the catalyst of adaptive immune system malfunction. The contributions of components outside the immune system, including environmental factors such as chronic viral infection also need more consideration, and much of the recent literature investigating the origins of this disease have focused on these factors. In this review, the details of the immunopathology of T1D regarding NK cell disfunction is discussed, along with how those mechanisms stand within the context of general autoimmune disorders. Finally, the rarer cases of latent autoimmune, COVID-19 (viral), and immune checkpoint inhibitor (ICI) induced diabetes are discussed as their exceptional pathology offers insight into the evolution of the disease as a whole.

## Introduction

Type 1 Diabetes (T1D) is a debilitating autoimmune disease that affects at least 1.6 million people in the US, accounting for ~5% of all diagnosed cases of diabetes, with an estimated 5 million people to be diagnosed by 2050 (1). Worldwide, of the ~463 million people living with diabetes, up to 10% have type 1 (2), representing an increasing incidence within an otherwise serious and increasing epidemic (3). It is well-established at this point that T1D results from autoimmunity, potentially involving both the innate and adaptive arms of the immune system. Indeed, most of the genes associated with greater risk in developing T1D point toward such an origin (4). However, the exact mechanism and the interplay between autoimmunity as well as the influence of environmental factors are still debated and investigated. The primary conundrum is that our understanding of these two components of the immune system is ever-evolving, and the conclusions made by studying *in vitro* or *in vivo* models like the non-obese diabetic mouse (NOD) or streptozotocin (STZ)-induced animals do not necessarily correlate one-to-one to the pathophysiology in humans. In addition, certain hypotheses involving multifactorial origins of T1D are difficult to test experimentally and frequently rely on correlative or epidemiological data rather than a discrete causality. It is likely that there are multiple etiologies and therefore, perhaps no two cases of T1D are the same necessarily.

At its core, T1D is a chronic autoimmune disease caused by destruction of the insulin-producing islet β cells, therefore rendering patients with the requirement of lifetime exogenous insulin supplementation (5). Oftentimes, diagnosis occurs at an early age, with clinical features indicative of hyperglycemia, such as increased thirst and frequent urination. Decreased circulating c-peptide levels and presence of autoantibodies, even prior to clinical manifestation portends the underlying immune-mediated attack. Early studies on autoimmunity focused on identifying autoantibodies and characterizing the pathogenesis, whereby autoreactive CD8+ T cells are the primary active immune cell in β cell death (6, 7). Islet autoantibodies for glutamic acid decarboxylase (GAD65), islet antigen 2A and insulin suggests a role for B cells, but to a lesser extent than the CD4+ T helper cells (e.g. Th1 and 2) thought to provide the pro-inflammatory cytokine profile necessary for activation of cytotoxic CD8+ T cells. More recent work suggests a more complete picture with innate immunity involvement – either in a destructive or regulatory role. Natural Killer cells (NK’s) are a bridge between the adaptive and innate arms of the immune system. They are capable of fighting pathogens or cancerous cells directly, and yet also generate memory cells and respond *via* antigen-mediated attack. They have long been associated with autoimmune diseases, and studies of their concentration, phenotype (frequency and function) and *in vitro* functionality in peripheral blood and tissue are numerous.

In this review, the immunoregulatory role of natural killer cells in the development of T1D will be presented, along with discussion of viral etiology, genetic risk, environmental factors, and even rare cases of T1D induced by cancer immunotherapy. The primary points of discussion will be the phenotypic character of pro-inflammatory and regulatory NK’s, their interplay with viral mechanisms of T1D induction in human and animal studies, and some alternative hypotheses involving late onset autoimmune diabetes and gut microbiome health that interweave nicely with the immunoregulatory role for NK’s. The central takeaway being the breakdown of self-tolerance that leads to T1D development is due ultimately to dysfunctional peripheral tolerance mechanisms associated with natural killer cells.

## Natural Killer Cells and Diabetes

Prior to the discussion of their role in the development of T1D, it would be prudent to briefly introduce the nature and function of natural killer cells (NKs), and to distinguish them from other immune cell subtypes that also play a critical role in the development of this disease, such as macrophages, T and B cells. Natural killer cells can be characterized as somewhat a hybrid between the innate or adaptive arms of the immune system. They mature from common lymphoid progenitor cells (CLP), recognize MHC Class I molecules, and exhibit targeted killing of virus-infected or transformed tumorigenic cells without prior sensitization *via* “missing self”-directed pathways (8, 9). They have a large cell body filled with cytolytic granules (perforin, granzyme B) similar to CD8+ effector T cells, but their activity is coordinated by a multitudinous array of both inhibitory and activating receptor-ligand interactions that can alter the NK cell status depending on levels of expression (9). It is conceivable that the evolution of NKs is a response to viral evasion of the adaptive immunity, thus giving rise to their innate phenotype with adaptive genotypic signature (10).

Distinct populations have been described for both mouse and human lineages, where they perform a Janus-type role of pleiotropic pro-inflammatory and regulatory functions (11, 12), somewhat analogous to the macrophage subtypes M1 and M2s (2a,b,c,d). They are the bone marrow-derived, thymus-independent third arm of the lymphocyte lineage that comprise 5-15% of peripheral blood mononuclear cells (PBMC’s) and take up residency canonically in the spleen and liver with small tissue-resident populations elsewhere (e.g. skin, liver, uterus). There are also subpopulations of NK cells that are capable of secreting anti-inflammatory cytokines (e.g. IL-10, IL-13, IL-27, TGF-β, IL-23) (13). Conversely, the more conventional populations can perform antigen-mediated cell lysis and apoptosis in addition to rapidly producing large quantities of inflammatory or directly cytotoxic molecules [principally IFN-γ (14), also TNF, GM-CSF, IL-5, IL-13, IL-22, and macrophage inflammatory proteins (MIP)] (15–17). Therefore, their role in immune system homeostasis is critical. Phenotypically, they carry quite a large array of distinguishing biomarkers, but in a simplified form, they are CD45+/CD3-, CD56+ (dim or bright), and CD16+/- depending on maturity. Some important receptors they carry involved in innate activation include the killer cell lectin-like receptors NKG2D and KLRG1, and natural cytotoxicity receptors (NCRs) NKp30, NKp44, and NKp46, while on the other hand receptors like the CD94/NKG2A dimer and the killer cell immunoglobulin receptors (KIRs in humans, Ly49 in mice) are usually inhibitory (18, 19). What is particularly interesting in the context of adaptive immunity, is how NK cells interplay with the activity of CD8+ cytotoxic T cells and CD4+ helper T cells (20, 21). It has been asserted that NKs act as initiators, mediators, and a hybrid of both, for which other reviews are available (22). Needless to say, they have a well-documented ability to prevent and control the CD8+ effector cytotoxic T cell response implicated in autoimmunity (21). As a results, they are frequently hypothesized as having an outsized role in the development of several autoimmune conditions (13, 23, 24), as they are the first in line in terms of developing an inappropriate response to a “self”-antigen or lack of sufficient presentation.

### Tissue-Resident Natural Killer Cells

The populations of NK cells resident within tissue (trNKs) (16) may possess more than just superficial phenotypic differences, perhaps even forming distinct lineages from NKs circulating within peripheral blood (cNKs) (16, 25, 26). The NKs that reside in the liver and skin are distinct from those in the blood and thymus, and from those that are intimately involved in preventing maternal rejection of the fetus within the uterus during pregnancy [uterine or decidual NK cells (16, 27–29)]. The exact function of these tissue-resident immune cells is unclear, but from an observational point of view, it is more nuanced than just cytotoxic mediators in the early stages of viral infection or tumor development and they are recruited for reasons outside of localized inflammation. It seems that they play a role in tissue homeostasis, and dysfunction or imbalances here could lead to several disease states, including autoimmunity. Resident NKs have been found in the pancreas of both diabetes-prone and normal mice (30), and possess an activated phenotype distinct from cNKs. It was also observed that they accumulate in the pancreas long before T cells and illustrate an exhausted and hyporesponsive state during later stages of disease (30). A similar effect was confirmed in a model for the autoimmune disease myasthenia gravis (EAMG) where the NK cells degenerated during the progression of disease and were mediated through an IL-21-dependent pathway by autoreactive CD4+ T cells (31). These observations are important to keep in mind during the interpretation of results from human studies where NK populations are decreased or non-functional.

### Natural Killer Cell Receptors and Their Ligands

The activity of natural killer cells is dictated by a balance between activating and inhibitory receptor-ligand interactions, some of which are immunoregulatory and therefore critical in the development of autoimmune disease. The NKG2D receptor is expressed by NKs among other immune cell subtypes in both human and mouse, binding to induced-self antigens of the MHC Class I polypeptide-related sequence (MIC) A/B which are overexpressed in infected (32) or otherwise transformed cells (e.g. tumorigenic) (33, 34). However, they have been reported to be constitutively expressed at low levels in many tissues including the pancreas (35). It is part of the greater NKG2 family of C-type lectin-like receptors. Unlike the CD94/NKG2A receptor dimer which also binds to MHC-I ligands (i.e. HLA-E), NKG2D is involved in activation/stimulation rather than inhibition and is costimulatory with CD8+ T cells (36). Effector status of NKs depends critically on the frequency and expression levels of this receptor (37, 38) and is therefore involved in regulating the activity of CD8+ T cells (36). The expression of NKG2D and, by extension, the activity of NK cells can be controlled by regulatory T cells (Treg) through a TGF-β mediated pathway (39), where Tregs are thought to down regulate its expression – leading to deleterious effects in the context of tumor surveillance but a pathway to understanding autoimmunity (40, 41). Although its ligands MIC A/B are normally expressed at sub-activating levels, NKG2D can accept a diverse array of ligands (42), one of which is retinoic acid early inducible 1 (RAE1, or ULBP in human), which is also constitutively expressed by pancreatic β cells and whose transcription is upregulated during viral infection in mice (25, 43, 44). This results in a precarious balance in the context of pancreatic trNKs, with both activating and inhibitory ligands being expressed constitutively. The activating NCR receptor NKp46 (NCR1 in mice) is considered especially important in the context of NKs and T1D since it is almost exclusively expressed by nearly all NKs (43, 45). Its function is also critical in terms of effective immunity toward viral infection, as noted by lethal influenza infection in NCR1 knockout mice (46). However, its ligands are yet to be fully characterized (47) and cross-reactivity toward molecular mimics is possible (45). Pancreatic β cells are thought to express from early development a yet unknown ligand for this receptor (48). This exposes these cells to potential NK attack if immunoregulatory/inhibitory receptors or ligands are insufficiently expressed. Regarding inhibitory receptors, the dimeric CD94/NKG2A serves an important role since it recognizes “self” antigens in the MHC-I family, including the non-classical HLA-E molecule. The expression of HLA-E is regulated by a complex set of processes but can be reduced or masked by some viral infections, which will be discussed more below. A related molecule in the non-classical MHC-I family is HLA-G, a ligand involved with immune protection/tolerance from NKs in the fetal trophoblast and anterior eye cell layers. It happens to be expressed by pancreatic β cells, which is hypothesized to be tied in with their insulin secretory activity as exocytosis exposes the extracellular space to myriad potential autoantigens (49). Its associated gene locus has naturally low-level polymorphism, suggesting small mutations could easily lead to a breakdown of immune tolerance, and there is some evidence from genetic studies correlating that region of the genome toward T1D susceptibility (50). Another set of inhibitory receptors in the killer cell lectin-like receptor subfamily (KLRs) include KLRG1 and KLRB1 (aka CD161) which are considered markers for activation (51) and senescent (52) phenotypes, respectively, but may play a role in regulating both cytolytic NK and T cell activity, potentiated by expression levels of its ligand lectin-like transcript 1 (LLT1) (53). The relationship between these receptors and T1D will be discussed at various points throughout the review. The importance of their role in disease etiology is a frequent point of contention, but regardless, they are ubiquitous throughout the literature.

### NK Observations in Humans

Early reports on NK cells from the peripheral blood mononuclear cells (PBMCs) of T1D patients showed a significant decrease in their proportion when compared to healthy individuals (54), which in one case was proposed as a possible explanation for higher occurrence of neoplastic tumors (54). Also among these early studies were reports from Negishi et al. that showed significantly decreased direct cytotoxicity *versus* relevant control samples against the K562 cell line with simultaneous increase in directed islet toxicity (55, 56). Some authors hypothesized aberrant NKG2D signaling in addition to decreased NK cell number as the primary driver for T1D development (57). However, the NKs present in peripheral blood only tell part of the story, as sequestered cNKs or potentially trNKs that are localized to the pancreas – where the important events unfold – may account for that deficit. Also, the functionality of these NKs from primarily long-standing patients may not be relevant to recent onset patients early in disease progression, as they are likely entering a ‘hyporesponsive’ phenotype (25, 31). The only consistent trend between NK populations and T1D in human patients is that the population in the peripheral blood is typically lower when compared to age/sex matched controls. In an analysis of the immune cell infiltrates of post-mortem pancreas samples of T1D patients, the most abundant cell type was CD8+ T cells, with very little NK detection (7). However, when an analysis of the tissue-resident immune cells of the pancreas of non-diabetic donors was performed (58), the majority of cells were also CD8+ T cells expressing markers for resident memory cells (CD69 and CD103). Here, NK’s represented only ~3% of the lymphocyte infiltrates. Given the similarity in distribution during healthy and diseased patients, it seems that what is being captured during this post-mortem examination may not be representative of critical phases in disease progression. That is where longitudinal studies such as those being carried out by the JDRF network of Pancreatic Organ Donors (nPOD) will be more revealing in terms of the evolution of immunophenotype at various time points along disease progression (59, 60). Other studies have stressed the importance of the natural cytotoxicity receptor (NCR) NKp46 expression on NK cells of diabetic patients (43, 61) which will be discussed from a mechanistic standpoint in animal models more below. In a study of isolated primary human islets, the presence of a ligand specific for the activating receptor was implicated in the NK cell mediated destruction of β cells, *in vitro*. It was found that the binding site on the receptor specific for its β cell ligand also binds viral and tumor associated proteins (48, 62). A takeaway lesson from these human studies is that the timing and nature of the sampling process is important when interpreting the results, as the peripheral blood cells of long-standing T1D patients may not provide the most accurate snapshot of the initial immune system alterations and dysfunction.

### Animal Model and Mechanistic Studies

Early studies on animal models yielded mostly conflicting results, albeit with some support of observed NK depletion (4, 63). For instance, a paper published in 1991 reported lower incidence of diabetes in a streptozotocin (STZ) mouse model when an NK specific antibody was administered before the first does of STZ, *versus* saline and non-specific Ig controls (64). However, just as early from Ellerman et al., it was demonstrated that in the BB/Wor rat model of diabetes, knocking down the population of peripheral NK cells with a 3.2.3 monoclonal antibody (mAb) did not prevent or delay diabetes onset, even though their critical role was hypothesized (63). Recent animal model work has demonstrated that after infection of rat insulin promoter RIP-GP mice with LCMV, induction of diabetes resulting from T cell activation (LCMV-gp) was regulated by NK cell levels and expression (20). Counterintuitively, the pancreatic tissue destruction was much worse in mice that were injected with low dosage virus (103 plaque-forming units) when compared to high dosage virus (65). The observed effect correlated with much greater NK cell activation and lower levels of tissue antigen-specific CD8+ T cells when high dosage of virus was used. When taken from high dose blood samples, those NK cells were directly cytotoxic toward autoreactive CD8+ T cells *in vitro*. The exact mechanism appears to be dependent on the expression of the NCR1 (NKp46) receptor in these NK cells, which was upregulated only in the case of high dosage, whereas expression of the receptor NKG2D was upregulated comparably in both viral dosages. A study in NCR1 knockout mice infected with LCMV confirms the observed mechanism of CD8+ T cell regulation (21). Strangely, this is in contradiction to previous observations in which NCR1 (NKp46) deficient mice were observed to have reduced T1D development (43), and where treatment of NOD mice with anti-NKG2D antibody prior to disease onset halted progression altogether (66, 67). Simultaneously, NKG2D ligands seem to be upregulated on target cells of diabetic model organisms (57). What role the NKp46 and NKG2D receptors play in T1D animal models is therefore a matter of contention, but may be resolved by considering the expression levels, the location of their respective ligands, the strength of the inhibitory signaling, and finally the longitudinal time of analysis, since NK effector status is always dictated by this balance. In the mice given high viral doses, the upregulation of NKp46 may have reflected its role in NK attack of CD8+ T cells with concomitant halting of disease progression. When β cell destruction is mediated through an antigen-specific process (aka after viral infection, discussed below), it follows that NKs targeting those T cells would inhibit that process. If there is an alternative pathway for the development of T1D, potentially *via* innate autoimmunity, the converse might be true, as in a case of NK activation *via* NKG2D/NKp46 ligand expression on β cells. The NKp46 receptor itself can be probed directly for its role in T1D development. Two separate studies from Mandelboim et al. showed that NCR1/NKp46 recognizes ligands expressed on mouse and human pancreatic β cells that specifically induce NK degranulation and subsequent cytotoxicity (43). Treatment of NOD mice *via* direct injection of a monoclonal antibody raised against the murine NCR1 receptor down-regulated its surface expression (68). This in turn led to a lower overall incidence in T1D development compared to appropriate controls, also observed in NCR1 knockout mice treated with STZ to induce diabetes (43). These animal models – while important for studying potential mechanisms – may lead to specious conclusions if the results from human studies are disregarded. Nonetheless, they still demonstrate the important role for NK activating receptors as well as their respective ligand interactions in both inflammatory and regulatory processes.

### Natural Killer T Cells

Not to be confused with natural killer cells, invariant natural killer T cells (iNKT) may also play a role in the autoimmune regulation and development of T1D (69). iNKT’s are tissue-resident innate-like immune cells whose defining quality is the expression of an invariant T cell receptor α-chain, and their recognition of CD1b. CD1b is an antigen-presenting molecule (MHC-I class-like) associated with dendritic cells (and some other APC’s) that displays lipid and glycolipid antigens of invading microbial pathogens. Although they do express cell surface markers of NK, such as CD161 (aka KLRB1) in humans, the expression of T cell receptors puts them distinctly into the latter class of immune cells descendent from the common lymphoid progenitor. They are primarily involved in defense against invading pathogens, tumor growth, and metastasis, but also play a regulatory role and can quickly release large amounts of cytokines like IL-4 and IFN-γ. Several studies using NOD mice have confirmed their effect on reducing the likelihood of diabetes development, which has been reviewed elsewhere (69). Suffice it to say, a similar effect as described above in the LCMV treated mice was also attributed to activation of NKT cells where they indirectly mediate CD8+ cytotoxic T cells *via* induction of TGF-β-producing Tregs (70). However, contradictory results in the number and type of NKT’s in human studies, in part due to very low number (~0.1%) in the peripheral blood and variable frequency in the general population makes it difficult to form definitive conclusions about their role in disease. This redundancy in the immune system reflects the hypothetical ease by which an autoimmune reaction could become problematic.

## Genetic Risk Factors and Autoimmunity

Although an auto-immune disorder of multifactorial origin, T1D does have associated genetic risk markers, suggesting a possible inherited risk. The observations of imbalance in population and aberrant behavior of NK cells in T1D patients certainly suggests a possible causal relationship in terms of islet cell destruction but this does not elucidate the related immune system malfunction, or, as in the case of viral infection, β cell susceptibility. Therefore, the associated genetic polymorphisms may be useful in identifying a link. Out of the >60 genes or loci that have been linked to a greater risk of developing T1D, the strongest correlations have been found with the HLA genes, specifically the class II alleles (71–74). This family of alleles is intimately involved in antigen presentation and recognition, a pathway involving APCs, B cells, and CD4+ T helper cells. Although adaptive immune response is important to the ultimate progression of disease, and abnormalities in the presentation of antigenic peptides by HLA molecules clearly may affect outcome, these correlations are not very useful in identifying the genetic role in the early precipitating events. This further supports the potentially larger role of environmental factors like viral infection relative to genetic predisposition towards a breakdown of central tolerance. It is likely that both are necessary for disease development with an environmental trigger that is amplified by genetic predispositions that manifest in defective immune response phenotypes. A fact supported by the tepid genetic linkage between T1D and other autoimmune disorders that are non-endocrine in origin (75). It has also been hypothesized that there exists a correlation between another allele, MHC Class I chain-related A (MICA), and risk for T1D, which similarly is involved in cell-cell communication and is a ligand for the activating receptor NKG2D. However, when a meta-analysis of ~5,000 patients with and without T1D was performed, variants of the MHC Class I chain-related A (MICA) were not found to be significantly correlated to T1D occurrence (76). Finally, the insulin molecule itself has been implicated in genetic predisposition (77), with some evidence to suggest CD8+ reactivity toward a pre-proinsulin epitope (78), which would fit in well with a disease progression that culminates with a primed adaptive immune system but still not explaining instigating events. In many of these studies, it is difficult to provide associative risk with absolute certainty due to the complexity in both the techniques used, and their accompanying analysis. However, emerging evidence in studies that look deeper than simple genetic mutation have revealed that even single nucleotide polymorphisms (SNPs) can alter how immunoregulatory genes are expressed (79), meaning the underlying genetic associations and/or susceptibilities have a complicated role in defining risk. Finding a concrete genetic link may be obscured underlying epigenetic factors that influence disease development. The role of microRNAs (or miRNA) in autoimmune disease in general has seen a tremendous surge in research effort (80, 81), and there is reason to suspect involvement in the development of type 1 diabetes (82). MicroRNA’s are involved in post-transcriptional regulation, in most cases silencing translation of target mRNAs, which in the context of autoimmune disease and T1D could mean a multitude of potential regulatory checkpoints. In addition, the discovery of circulating miRNAs associated with T1D could lead to their use as biomarkers for early detection or to identify at-risk individuals (83). Among the profile of miRNAs identified in exosomes isolated from human blood samples in one study, seven were differentially expressed in patients with diabetes (84).

## Viral-Mediated Type 1 Diabetes

Viral infections are hypothesized to be involved in myriad autoimmune diseases (85, 86). As alluded to above, viruses play a critical role in the onset or potential for acquiring T1D reflected by their prevalence in animal model studies (87, 88). Their use in eliciting the understanding of disease progression with regard to NK cells is invaluable, as the two are inexorably linked (28, 89). The etiology suggests that enteroviruses (90, 91) (e.g. CV-B4) or those belonging to the Herpes family (92, 93) are the most likely contributors in humans, as many recent-onset patients show enteroviral nucleic acid or other viral biomarkers like viral capsid protein and IgM indicative of recent infection. Conversely, the prevalence of viral biomarkers in control populations of healthy individuals is significantly lower (88, 90, 94, 95). Due to improved detection and sampling methods, only recently has a definitive link been established (96, 97). However, it is not completely clear whether the presence of virus indicates causality or is a result of diminished ability to fight off viral infection due to reasons like suppressed/altered NK levels or dysfunctional adaptive immunity. The role for virally-mediated development of T1D has been reviewed in great detail elsewhere (89, 98–101), and therefore only the relevant material will be discussed here.

### Viral-Mediated β Cell Destruction

**Figure 1:**
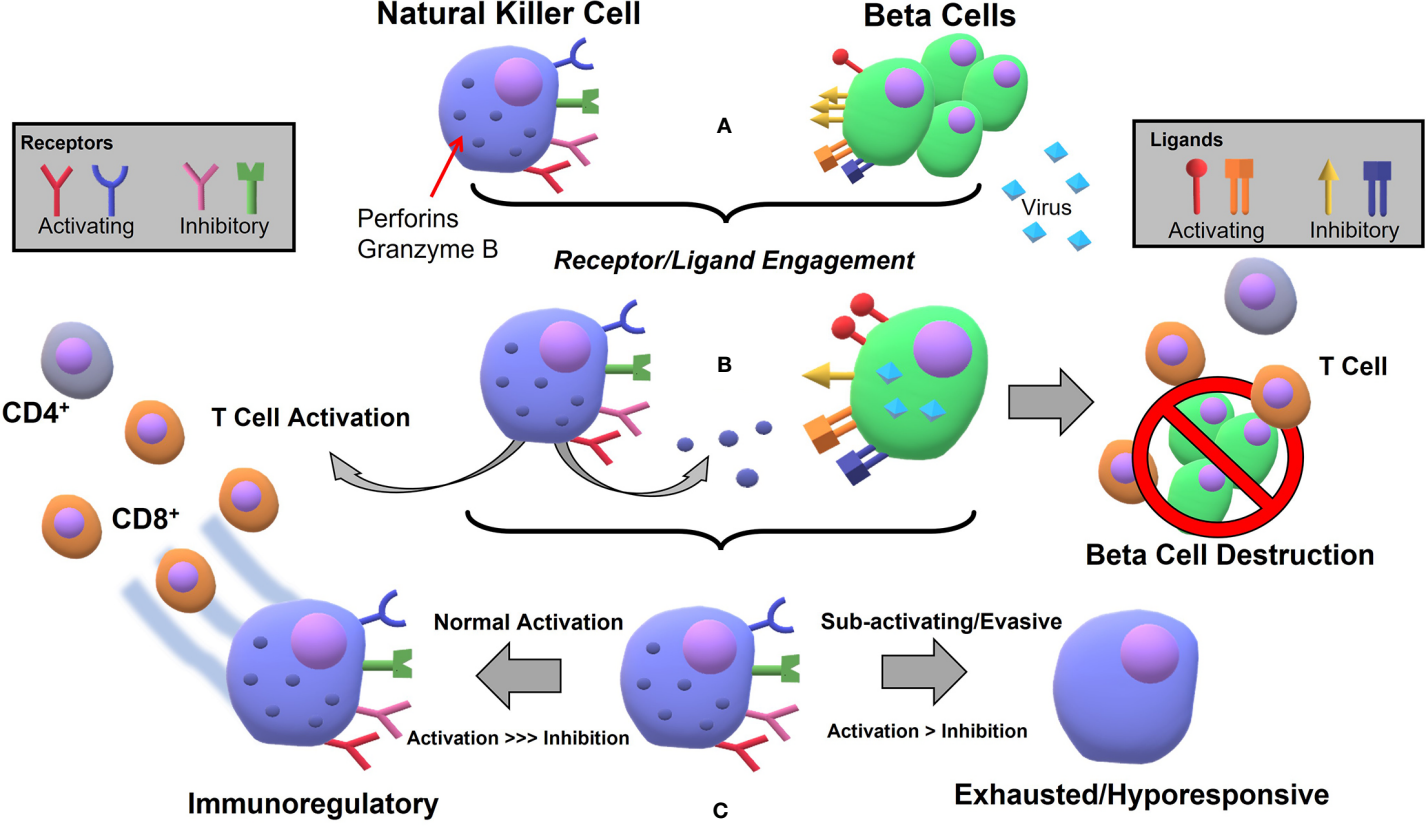
Hypothetical process for NK mediated β cell destruction and subsequent autoimmune T cell reaction following viral infection in pancreatic tissue: **(A)** Natural Killer cells express several activating (NKp46, NKG2D, some KIRs) and several inhibitory (CD94/NKG2A, KLRG1, KLRB1, some KIRs) receptors for ligands which can be expressed at varying levels on β cells during normal stasis. Ligands include the inhibitory set of MHC-I molecules (e.g. HLA-E) and the activating inducible MIC A/B molecules, constitutively expressed RAE-1/ULBP, and the unknown ligand for NKp46 **(B)** Viral infection leads to NK activation, cytokine release, whereby adaptive immunity is recruited and NKs degranulate, killing infected cells **(C)** If the viral infection is at low-level, persistent, or the virus is able to use evasive or immunosuppressive tactics, the NKs will not react appropriately and the immunoregulatory feedback does not occur, leading to exhausted or hyporesponsive state and β cell destruction by autoimmunity.

The general mechanism by which viral infection can lead to autoimmune disease is assumed to be the following: (1) infection localized to some target organ first activates an innate immune response (e.g. macrophages, NKs) (2) those cells become cytotoxic toward the autologous infected cells causing tissue damage beyond what is sufficient to clear infection (3) subsequent antigen release/processing recruits an already primed adaptive immunity in a runaway inflammatory cascade leading to lasting or permanent damage of the tissue/organ. Given the right mix of genetic risk and environmental factors, the process can easily lead to an autoimmune disease state. Viruses have evolved countless ways to outsmart adaptive immunity designed to seek them out *via* modulation of the expression of MHC class I peptide complexes (102, 103), which underlines how important NK cell function is. NKs are the principal defenders against viral invaders, secreting copious IFN-γ and inducing cytotoxicity in infected cells without the need for a priming phase *via* the “missing-self” mechanism. This leads to one hypothesis being that defective NKs result in viral-induced T1D development, and that process can go one of two ways. In the “pro-inflammatory” defective state, NKs are far too aggressive in viral clearance and T cell recruitment. In an “immunosuppressive” defective state, NKs do not respond appropriately to viral infection, allowing for chronic or persistent infection and/or β cell destruction by uncontrolled cytotoxic adaptive T cells. As evidence for the pro-inflammatory defect, one study showed that type I interferon (IFN-1) transcriptional signatures are associated with an increased activated innate immune response in patients pre-disposed to developing T1D, and confirmed after a longitudinal study that those with the strongest signature went on to develop the disease (104). One of the genes identified with increased T1D risk, IFIH1, encodes the MDA5 receptor that recognizes viral RNA and induces IFN-1 signaling. Reduction of that receptor by >50% (using a IFIH1 knockout) on an NOD mouse model protected them from T1D development without diminished ability to clear virus (105). Arguments for the overly immunosuppressive side have been put forth as well. In their normal regulatory capacity, NKs secrete IL-10, which has been observed to play a role in immunosuppression during systemic infection but less so local infection (106). An infection localized to the pancreas would be unlikely to induce such expression, but it has been hypothesized that infected islet cells can secrete IL-10 to avoid extensive T cell recruitment (90). Perhaps in the context of β cell infection and subsequent insulitis, NK cells are not appropriately activated and do not secrete sufficient IFN-γ to recruit effector CD8+ T cells to efficiently clear virus, which has been demonstrated in a recent study of RIP-GP mice at low levels of viral infection (20). It would seem then that counterintuitively, NK cells in T1D development are defective on two fronts – simultaneously attacking β cells and producing pro-inflammatory cytokines that lead to T cell recruitment, while unable to clear infection which allows for persistent and destructive insulitis. Another hypothesis which has been proffered could better explain this etiology, centered on the reasoning that viral modulation of the immune response causes defective NK-signaling. For example, β cells could be particularly susceptible to specific viruses leading to pervasive infection and improper clearance (65), or chronic infection and immune-evasive tactics of some viruses may ultimately lead to destruction, as might be expected for viruses undergoing lysogenic-lytic cycles. One interesting hypothesis that has emerged is the role of reactivated human endogenous retrovirus (HERV), whereby environmental or inflammatory stimulus (e.g. other viral infection) allows for activation of HERV transcription and gene expression that could once again either cause direct damage to islet cells, or induces an autoreactive immune response by affecting activating or inhibitory receptor-ligand interactions (107). A hypothesis that might aid in the understanding of this viral-mediated process is the following (**Figure 1**). In a normal response, sentinel pancreatic NK cells take on a regulatory phenotype after the initial phases of innate activation leading to effector status toward CD8+ T cells, thereby preventing β cell destruction and T1D. However, in a dysfunctional response, one of two things (or combined effect) occurs. Either (1) NKs become exhausted/hyporesponsive, diminish in activity and number, and allow for what is typically understood as the major mechanism for β cell destruction, aka CD8+ T cell autoreactivity, (2) Viral pathogens hijack mechanisms for immune modulation (like over-expression of HLA-G and modulation of HLA-E) thereby turning NK cells into a suppressive force that allows the adaptive response to go unchecked. The regulatory or immunosuppressive capacity of NKs has been demonstrated in both systemic (106) and local (20, 21) infection, and it stands to reason that dysregulation at this junction could be a deciding factor in T1D development, perhaps reconciling the observations of impaired T regulatory ability as well (108).

### Sars-CoV-2 and Diabetes

Considering the recent pandemic, it would be appropriate to examine the recent cases of T1D following COVID-19 infection. Diabetes, especially type II, has been established as an associated increased risk factor for developing severe disease, but does the SARS-CoV-2 virus itself present as a possible cause of diabetes? There have now been more than merely isolated cases of hyperglycemia, lasting β cell damage and other severe metabolic complications in COVID-19 patients, in some cases remitting after a few weeks, but in others developing into lasting disease (109, 110). The virus enters the cell *via* the angiotensin-converting enzyme 2 (ACE2), a receptor expressed on multiple cell types, including endocrine cells of the pancreas, making SARS-CoV-2 a plausible case for COVID-19 induced diabetes. Indeed, it is documented that coronaviruses can cause multi-organ damage by entering through these receptors (111), and *in vitro* pancreatic-like organoids derived from induced pluripotent stem cells are susceptible to viral entry *via* a spike-protein mediated attack (112). Curiously, in comparison to the other cell types generated, the pancreatic organoids were much more permissive to viral entry. In an analysis of post-mortem COVID-19 patient samples and ex vivo islets, the presence of SARS-CoV-2 protein colocalized with the NKX6.1 β cell marker was confirmed. Interestingly, infection elicited an interferon transcriptional signature reminiscent of that which proceeds T1D (104, 113). Studies on the links between the latest coronavirus and new onset diabetes are nascent and ongoing, and it remains to be seen if and how it fits in with the analysis presented here.

## Unique Cases

### LADA

Latent autoimmune diabetes in adults (LADA) is characterized by a pathological state that is clinically defined as having characteristics of both Type 1, with the presence of autoantibodies (GAD), and Type 2 diabetes with typically – though not always – later onset (114, 115). In simple terms, age, and insulin dependence at the time of diagnosis are considered critical factors. However, LADA can be viewed as a milder or slower moving case of T1D since autoantibodies and β cell reactive T cells are still present and exogenous insulin supplementation is usually required. Therefore, the disease allows for careful longitudinal study of the progression of autoimmune diabetes. As with recent onset T1D patients, individuals with recently diagnosed LADA exhibit a decrease of NKs in the peripheral blood when compared to healthy individuals (116). In one case of recently diagnosed LADA patients, however, it was reported that NK frequency increased, especially of activated NKp46+ cells (61). The expression of the activating receptor NKG2D and inhibitory receptor KIR3DL1 was increased and decreased in these patients, respectively, and a reduced frequency of CD4+CD25+ T regulatory cells was observed (116, 117). Notably, a lower proportion of APC’s and higher number of regulatory B cells (IL-35+) was observed in LADA patients when compared to healthy control and T1D patients (118). These combined observations lead to another important inference about the phenotype of those with this form of the disease. The immune cells and their receptors that are ultimately responsible for activating/regulating the β cell destructive CD8+ cytotoxic T cells are decreased/increased, respectively in LADA compared to T1D. However, they are still increased/decreased compared to healthy controls, representing an intermediate immunophenotype. Whether this observation is a result of disease pathology or is a causal agent has not been elucidated. Still the correlation supports the notion that the cytotoxic CD8+ T cell “finishes the job” after recruitment to the target organ *via* NK-mediated pathways. It has been hypothesized that the CD4+CD25+ regulatory T cells regulate NK cells’ NKG2D expression *via* a TGF-β-dependent pathway. A disruption of said pathway may lead to the upregulation of this activating receptor (40, 41). If this inhibitory signaling is outpaced, a clear imbalance results. In developing cases of latent diabetes, therefore, a treatment to prevent total islet destruction may be possible. For instance, the monoclonal antibody drug Monalizumab, which targets the inhibitory natural killer cell receptor NKG2A, is currently under clinical investigation for use in the treatment of some cancers and autoimmune conditions like RA (119). An analog targeting the NKG2D receptor may be useful in terms of preventing the full transition to insulin dependent type 1 diabetes when administered early in disease progression. In a clinical trial evaluating hematopoietic stem cell transplantation to treat T1D, patients that required lower exogenous insulin saw increased TGF-β and IL-10 immunoregulatory and decreased IFN-γ, IL-2 inflammatory cytokines (120).

### Immune Checkpoint Inhibitor Diabetes

ICI chemotherapy (immunotherapy) is a recently approved cancer treatment (121), but there are non-phenomenological case reports and clinical reviews that definitively demonstrate immune-related adverse events (IRAEs) leading to the development of diabetes or at the very least diabetic ketoacidosis (122–125). Although the cause of diabetes or other autoimmune side effects in these cases does not coincide with the paradigm of normal pathogenesis of the disease, it is worthwhile to briefly examine how the two are related, especially within the context of participating immune cells. In the case of programmed death-1 (PD-1) inhibitors, the prevailing therapy associated with these outcomes (124), their mechanism of is to bind to the transmembrane protein located on the surface of activated T cells in order to block the “hand-shake” interaction with its associated ligands, PDL-1/2. This interaction limits autoimmunity during inflammatory responses. As a result, activated T cells can directly target the proliferating tumor cells, and to the detriment of a very small number of patients (~1%) act upon the β cells of the pancreas leading to diabetes development. It is possible the susceptibility is genetic and related to altered or lowered PD-1 expression that is also observed in T1D patients (126–128). However, the fact that these patients who developed diabetes only after immunotherapy treatment were of relatively advanced age, and in many cases had disease reversal upon cessation of treatment suggests that the PD-1 related susceptibility is not sufficient in and of itself for developing disease. Once again, a role for regulatory immune cells, like the NKs alluded to above, may prevent this effect from being more common among ICI patients and treatments utilizing transformed NKs are becoming more acceptable (129).

### Gut Microbiome

Although the implications of the health of gut microbiota sometimes stretch further than what is empirically proven, it is obvious that there is a potential role for the microbiome in autoimmunity and even the development of T1D. Several reviews poring over the mechanistic and genomic details that underpin the relationship between the two are available (130–132). For the sake of brevity, we highlight a few important studies that complement the pathology described above. Some early evidence exemplifying the role of environment and microbiome in animal model studies was that the incidence of NOD mice developing diabetes is drastically increased when raised in completely “germ-free” environmental conditions (133, 134). In a separate study, NOD mice given an intraperitoneal administration of a bacterial extract containing a cocktail of bacteria that cause respiratory tract infections either prevented or delayed the onset of disease (135). The effect was neutralized by administration of anti-TGF-β antibody, suggesting a role for and potential increase in concentration of this regulatory cytokine after extract administration. It was suggested that the pathway would involve NKT cells, but Cd1d-/NOD mice did not show much difference in their response. As mentioned above and in ref (40), it is thought that TGF-β mediates the expression of NKG2D on natural killer cells, naturally modulating their innate immune activity toward potentially infected or transformed cells. This suggests the extract may supplement natural TGF-β production needed to suppress NKG2D receptor activation, attenuating NKs that may otherwise target β cells. Further study should target NK deficient animal models instead to pin down the culprit immune cell(s).

## Discussion

The development of autoimmune diabetes is generally thought to progress as follows. A susceptible person has at most minor abnormalities in the number and phenotype of immune cells such as NKs as a result of genetic and/or environmental factors (e.g. microbiome activity, endogenous virus, epigenetic regulation). An external stimulus – most likely viral infection – is key in precipitating a peripheral immune reaction that leads to formation of autoreactive T cells and antibodies that ultimately leads to the destruction of the functional pancreatic islet cells and necessitation of insulin dependence. NKs are involved at an early stage, where external stimulus takes place and peripheral tolerance breaks down. The receptors as well as their ligands that are involved in NK activation are both aberrantly activated, and β cell attack becomes inevitable. It should be emphasized that this represents the collapse of a very fragile balance, where the combination of several small factors exponentially precipitates into a catastrophic event. The existence and rarity of late-onset autoimmune diabetes exemplifies this fact, as avoiding the confluence of these small events late into adulthood is highly unlikely.

## Implications for Treatment and Concluding Remarks

In many of the studies discussed above, the natural assumption can be made that only preemptive surveillance and hypervigilance would make it possible to prevent the development of T1D. After the initial signs of β cell loss, it seems that there is little that can be done to reverse its course outside of auto/allo-transplantation of functional tissue under the blanket of systemic immunosuppression. Unfortunately, that limits the clinical reach of T1D treatment to patients with severe hypoglycemic unawareness. Refinements in donor islet and stem-cell derived tissue implantation have come a long way and increased the available tissue source. Additionally, there is a concerted effort to eliminate systemic immunosuppression with efforts targeting localized delivery of immune modulatory agents coupled with immune evasion through encapsulation and/or genetic manipulation. While currently in their infancy, immune cell therapies could one day play a role as well, still requiring further study before clinical applications could be explored. Through a detailed study and understanding of the progression to T1D onset, it may be possible to develop prevention strategies without undue burden of painstaking surveillance. Routine genetic screens are now commonplace for many hereditary diseases and adding another T1D-specific panel would not be prohibitively costly. Also, with emerging scientific consensus on the importance of a healthy gut microbiome as an environmental factor, strategies to improve gut health would be easy to implement.

Although it is still a subject of ongoing investigation, the defining picture of T1D autoimmunity is becoming clearer, albeit perhaps more complex than originally thought. Conflicting results that arise from a limited pool of samples, sample selection, stage of disease, etc, and inappropriate *in vitro* or *in vivo* models have confounded progress. However, recent research efforts to expand sample availability and collaborative efforts, such as the JDRF-nPOD, have accelerated discovery on many fronts. As it stands today, it is becomingly increasingly obvious that the progression of T1D occurs because of improper activation and dysregulation of the immune system starting with natural killer cells and viral infection. In this review, we focused on the topic from the standpoint that the primary breakdown occurs at peripheral immune tolerance, as brought out by a dysfunctional set of primarily pro-inflammatory natural killer cells that precipitates the adaptive response and auto-immunity characterized by the disease. The reason for this breakdown is hypothesized to be a combination of the overexpression of activating receptors/ligands ascertained from genetic risk factors, lack of immunosuppressive support from the microenvironment, a likely viral triggering event. For the next steps, a method by which to recognize the early signs of this action and slow or halt its progression will be an ideal treatment to put an end to this pandemic.

## Author Contributions

CF and GG contributed to the conception of the manuscript. GG wrote the original draft. GG, and CF contributed to the manuscript revision and editing. All authors contributed to the article and approved the submitted version.

## Funding

Research reported in this manuscript was supported by funding from the Diabetes Research Institute Foundation.

## Conflict of Interest

The authors declare that the research was conducted in the absence of any commercial or financial relationships that could be construed as a potential conflict of interest.

## Publisher’s Note

All claims expressed in this article are solely those of the authors and do not necessarily represent those of their affiliated organizations, or those of the publisher, the editors and the reviewers. Any product that may be evaluated in this article, or claim that may be made by its manufacturer, is not guaranteed or endorsed by the publisher.
